# Resin Composite Injection Technique With a Digital Workflow To Reconstruct Canine Guidance: A Two-Year Follow-Up

**DOI:** 10.3290/j.jad.c_2213

**Published:** 2025-08-18

**Authors:** Yuta Utsumi, Keiichiro Watanabe, Sooha Matsuki, Takuma Sakamaki, Eiji Tanaka, Keiichi Hosaka

**Affiliations:** a Yuta Utsumi PhD Student, Department of Conservative Dentistry, Tokushima University Graduate School of Biomedical Sciences, Tokushima, Japan. Clinical Fellow, Section of Implant and Rehabilitative Dentistry, Division of Oral Rehabilitation, Faculty of Dental Science, Kyushu University, Fukuoka, Japan. Concept and design, wrote the manuscript; acquisition, analysis, or interpretation of data.; b Keiichiro Watanabe Assistant Professor, Department of Orthodontics and Dentofacial Orthopedics, Tokushima University Graduate School of Biomedical Sciences, Tokushima, Japan. Concept and design, Critical review of the manuscript for important intellectual content, acquisition, analysis, or interpretation of data.; c Sooha Matsuki Assistant Professor, Department of Orthodontics and Dentofacial Orthopedics, Tokushima University Graduate School of Biomedical Sciences, Tokushima, Japan. Acquisition, analysis, or interpretation of data.; d Takuma Sakamaki Dentist, Ohki Dental Clinic, Mie, Japan. Acquisition, analysis, or interpretation of data.; e Eiji Tanaka Professor, Department of Orthodontics and Dentofacial Orthopedics, Tokushima University Graduate School of Biomedical Sciences, Tokushima, Japan. Critical review of the manuscript for important intellectual content.; f Keiichi Hosaka Professor, Department of Conservative Dentistry, Tokushima University Graduate School of Biomedical Sciences, Tokushima, Japan. Visiting Professor, Smart Innovations for Preventive and Restorative Dentistry Research Group, Department of Operative Dentistry, Faculty of Dentistry, Chulalongkorn University, Bangkok, Thailand Adjunct Lecturer, Division of Operative Dentistry, Department of Ecological Dentistry, Tohoku University Graduate School of Dentistry, Sendai, Japan. Adjunct Lecturer, Institute of Science Tokyo, Tokyo, Japan. Concept and design, Critical review of the manuscript for important intellectual content, and supervision.

**Keywords:** clear index, digital workflow, direct resin composite injection technique, pragmatic esthetics, tooth wear

## Abstract

**Purpose:**

Orthodontic treatment often requires anatomical tooth reconstruction for esthetic and functional reasons. Direct resin composite (RC) restorations, which preserve tooth structure while providing high bond strength, are widely used. Recently, orthodontic treatment plans increasingly incorporate RC restorations for correction of tooth form. This report presents a case in which RC injection, aided by a digital workflow, successfully restored lost canine morphology.

**Materials and Methods:**

An intraoral scanner was used to obtain optical impressions and occlusal records, which were then imported into a computer-aided design (CAD) system for analysis using a virtual articulator. Lateral movements were simulated in the virtual articulator to replicate occlusal contacts. Subsequently, a digital wax-up was used to fabricate a 3D-printed model, and a transparent silicone index with designated openings for RC injection was created. A flowable RC (Filtek™ Supreme Ultra Flowable Restorative, Solventum) was injected through the incisal access opening of the index.

**Results:**

By utilizing a clear index designed through the digital workflow, the wear of the canine teeth was efficiently restored using the injection technique. Evaluation of occlusal guidance with a digital articulator revealed that minimal morphological adjustment was required, resulting in a significant reduction in chair time. At the two-year follow-up, the clinical outcomes remained highly favorable.

**Conclusions:**

Preoperative occlusal simulations enable precise transfer of tooth form, enhancing clinician-technician communication and reducing chair time. Despite requiring additional visits for index fabrication and specialized equipment, digital workflow-assisted RC injection guarantees esthetic and functional outcomes.

In recent years, the concept of minimal intervention dentistry (MID) has gained considerable attention, and the use of dental adhesives and resin composites (RCs) as effective approaches to address esthetic concerns has been widely recognized.^10,16^ In particular, restorations utilizing RCs with high bond strength allow for minimal tooth structure removal, contribute to reduced treatment time and risk of complications, and thus serve as a clinically effective treatment modality.^1,2,5^ Moreover, recently introduced universal shade RCs have enhanced shade-matching capabilities, allowing for more esthetically pleasing outcomes.^8,22^ As a result, RC restorations – which aim to preserve tooth structure while achieving favorable esthetic results – have been applied in a wide range of clinical situations, including treatment of tooth wear, prosthetic rehabilitation of missing teeth, optimizing tooth form, anterior restorations, and correction of malocclusions.^3,7,10^ Following orthodontic treatment, it is common to perform restorative procedures to correct tooth form for both esthetic and functional purposes.^13^ Consequently, orthodontic treatment planned in combination with correction of tooth form using RC restorations has become increasingly common in recent years.

Traditionally, adhesive porcelain restorations and zirconia prostheses have been employed as treatment modalities for the correction of malocclusion. Due to the high level of technical skill required for direct freehand RC restorations, especially in cases where esthetic demands are high, there is a prevailing tendency to select indirect restorations that can be fabricated extraorally, despite their relatively greater invasiveness compared to direct techniques.^4^ Furthermore, such procedures have been reported to be associated with longer chairside time, higher costs, and increased patient discomfort.^1^

To address these challenges, an innovative approach has been proposed involving the use of a pre-fabricated clear silicone index for the injection of flowable RC.^9,18^ This RC injection technique utilizes a transparent index as a mold, allowing for reduced chairside time and the delivery of predictable esthetic outcomes. Although this method requires preliminary laboratory procedures, recent advances in digital technology have significantly improved its accuracy and reliability, allowing a seamless transition from the digital wax-up to the final restoration.^4,16,20,21^ Moreover, the use of scanned data for articulator mounting and occlusal simulation has made it possible to approximate and replicate functional mandibular movements.

This case report presents the restoration of esthetics and function of the maxillary and mandibular left canines affected by moderate incisal wear using the principles of MID and incorporating digital workflow technologies. The RC injection technique, using a digital wax-up and a clear silicone index, was applied to achieve precise restoration of the worn canines with careful consideration of occlusion and articulation.

## MATERIALS AND METHODS

### Case Presentation

The patient in this case was a 22-year-old male who presented with chief complaints of dissatisfaction with the appearance of his teeth and difficulty chewing. Clinical examination revealed maxillary protrusion and anterior crowding, for which orthodontic treatment was required (Fig 1a, b). Accordingly, treatment with a multibracket appliance was initiated at the Department of Orthodontics (Fig 1c, d). Moderate incisal wear of the maxillary and mandibular left canines due to malocclusion was managed with a treatment plan that included reconstruction of form and function with RCs (Fig 2). For this latter treatment, the patient was referred from the Department of Orthodontics to the Department of Conservative Dentistry for restoration of the morphology of the maxillary and mandibular left canines.

### Fabrication of a Clear Silicone Index Using a Digital Workflow

An optical impression was taken using an intraoral scanner (IOS) (Primescan; Dentsply Sirona), and the occlusal relationship was recorded in maximum intercuspation. For the digital wax-up, the anatomical morphology of the left maxillary canine was designed using CAD software (Dental System; 3Shape) (Fig 3a, b). The design was subsequently mounted on a virtual articulator, which allowed three-dimensional evaluation of the crown morphology and occlusal structure, along with occlusal stress analysis based on the scanned data (Fig 3c). A functional wax-up was then performed, incorporating lateral movements to establish appropriate canine guidance (Fig 3d, e).

The 3D model data were optimized using modeling add-on software (Model Builder; 3Shape), and final models of both arches were fabricated using a high-resolution stereolithography 3D printer (DigitalWax 028D; DWS) (Fig 4a). A transparent polyvinyl siloxane material (EXACLEAR; GC Corp.) was applied to the model, and polymerization was carried out under a pressure of +0.2 MPa for 10 min using a pressure molding device (Erkopress; Smart Practice) (Fig 4b). This process ensured high transparency and rigidity of the index while minimizing bubble formation.

Finally, an access opening for RC injection was created at the incisal edge of the index by inserting the tip of an RC syringe, thereby completing the preparation of the clear silicone index (Fig 4c).

### Clinical Procedure of the RC Injection Technique for the Maxillary Left Canine

Following rubber dam isolation, the tooth surface was cleaned with a prophylactic paste (Presage; Shofu) (Fig 5a). Enamel etching was performed with 37% phosphoric acid (Scotchbond Universal Etchant; Solventum), followed by the application of a universal adhesive system (Scotchbond™ Universal Plus Adhesive; Solventum). A metal matrix band was placed to prevent the adhesive from inadvertently contacting the adjacent tooth surface.

To block the flow of RC into the gingival embrasure, polytetrafluoroethylene (PTFE) tape was gently packed into the interproximal space. The clear silicone index was then positioned accurately, and a low-viscosity flowable RC (Filtek™ Supreme Ultra Flowable Restorative, Solventum) was injected through the incisal access opening of the index (Fig 5b). Light curing was performed for 10 s using an LED curing unit (Pencure 2000; MORITA), after which the index was removed and an additional 10-s light curing was carried out to ensure complete polymerization.

### Clinical Procedure of the RC Injection Technique for the Mandibular Left Canine

Due to the presence of a fixed retainer extending to the canine region, moisture control was achieved using a combination of a splitdam (Optradam; Ivoclar) and a salivary absorbent device (DryDent; DIRECTA) (Fig 6a). Following isolation, the tooth surface was cleaned with a prophylactic paste (Presage; Shofu), and the enamel was etched with 37% phosphoric acid (Scotchbond Universal Etchant; Solventum). A universal adhesive system (Scotchbond™ Universal Plus Adhesive; Solventum) was then applied to the tooth surface.

To prevent unintentional adhesion of the adhesive system to the adjacent tooth, the neighboring tooth was covered with PTFE tape. Additionally, a separating agent (Washable SEP; Sun Medical) was applied to the adjacent surface to further prevent adhesion of the RC to the neighboring tooth. After ensuring proper placement of the silicone index, a flowable RC (Filtek™ Supreme Ultra Flowable Restorative; Solventum) was injected through the incisal access opening of the index (Fig 6b). Light curing was performed for 10 s using an LED curing unit (Pencure 2000; MORITA), followed by index removal and an additional 10-s light-curing cycle to complete polymerization.

### Finishing and Polishing Protocol Following RC Restoration

Under a dental operating microscope, excess RC was carefully removed using a #11 surgical blade. Occlusal adjustment and final polishing were then performed (Figs 4c, 5c). A two-step polishing system (FP9769M, FP9769F Meisinger Polisher; Hager & Meisinger) was utilized in combination with a polishing brush embedded with abrasive particles (OptiShine™; Kerr). This technique allowed for minimal adjustment and significantly reduced chairside finishing and polishing time.

## RESULTS

At the two-year follow-up, the clinical outcome remained favorable with no signs of complications or deterioration (Fig 7a, b). A slight, clinically acceptable marginal discoloration was observed on the mandibular canine; however, it was successfully removed by polishing (Fig 7c, d). Overview of the dentition demonstrates a well-balanced restoration on both sides, with satisfactory esthetic and functional outcomes for the patient (Fig 8). Quantitative evaluation was performed using IOS data obtained at one and two years postoperatively. The measurements were taken at the areas that appeared to exhibit the most wear, based on a comprehensive assessment of the cross-sectional views obtained from the IOS data at one and two years postoperatively. The analysis revealed a difference of approximately 0.2 mm in the maxillary canine and 0.04 mm in the mandibular canine (Fig 9).

## DISCUSSION

RC restorations that minimize the removal of sound tooth structure are widely recognized as clinically effective, contributing to reduced treatment time and less invasive procedures.^9,17^ As such, RC has been used in a wide range of clinical scenarios – including the restoration of defects, correction of tooth color, form, and position, anterior restorations, and improvement of mild malocclusion – with a focus on tooth preservation and esthetic outcomes.^3,10^

However, recreating the form of the tooth that is both esthetic and functional through a freehand technique is not straightforward. Freehand RC restorations are highly operator-dependent and may increase chairside time.^1^ To overcome these challenges, the RC injection technique using a clear silicone index and highly filled flowable RC offers a predictable and efficient solution. This technique allows for precise preoperative planning and simulation, which can then be accurately transferred to the clinical setting, leading to improved restorative outcomes.^4,14,20,21,23^

Additionally, highly filled flowable RC materials have demonstrated comparable mechanical strength, wear resistance, esthetics, and long-term durability to conventional RCs, supporting their reliability in anterior esthetic applications.^5,11^ The use of single-shade injectable RC also streamlines the restorative process by eliminating the need for shade matching and incremental layering, while still achieving satisfactory color integration and natural esthetic blending with the surrounding dentition.^8,22^

In the present case, the use of an optical scanner enabled the digital acquisition of clinical records, allowing for CAD-based design of the restoration. The scan data were imported into CAD software, where the anatomical, esthetic, and functional morphology of the tooth was precisely designed. Additionally, virtual articulation allowed the simulation of lateral movements, which facilitated the reproduction of appropriate occlusal guidance in the clear silicone index. This digital workflow enabled effective communication and morphological consensus between the clinician and the dental technician, ensuring accurate transfer of the planned design – including canine guidance – into the clinical outcome. As a result, minimal adjustments in form and occlusion were required, and the restoration could be completed with minimal polishing, contributing to reduced chairside time.

Nevertheless, this approach has certain limitations. The need for additional appointments to fabricate the index, as well as the requirement for specialized equipment and software, can pose practical challenges. The cost of this type of treatment will for sure be higher due to the need for specialized equipment, digital tools, and trained personnel.

However, given that freehand RC restorations are technique-sensitive and often lack predictability and efficiency, digital workflows offer significant advantages by reducing operator dependency and ensuring consistent, minimally invasive, esthetic, and functional outcomes. While indirect prosthetic restorations typically offer superior mechanical strength and wear resistance compared to direct RCs, they often require more extensive removal of healthy tooth structure and may increase the risk of pulpal irritation. From the perspective of MID, direct RC restorations have the advantage of preserving healthy tooth structure and minimizing pulpal trauma.

It should be noted, however, that the flowable RC used in this case may demonstrate inferior wear resistance compared to paste-type RCs, as reported in previous studies,^14,19^ and there are concerns regarding material degradation in the intraoral environment over time.^24^ For this reason, the use of occlusal splints is considered effective in mitigating wear,^15^ and regular clinical follow-up is essential. An alternative approach involves preheating paste-type RCs to enhance their flowability, thereby enabling their use in injection techniques.^6^ However, challenges remain in the uniform delivery of such materials within the index, and concerns such as potential index displacement during injection highlight the need for further refinement of the technique. Moreover, in the initial protocol, the clear silicone index involved only a single injection access hole, with excess material management relying on visual confirmation of overflow at the margin. To improve control over excess material and enhance the predictability of the injection process, a modified technique incorporating an additional escape (ventilation) hole was subsequently developed.^12^

Although no significant issues with anatomical morphology or color were observed at the two-year follow-up, a slight, clinically acceptable marginal discoloration was observed on the mandibular canine; however, it was successfully removed by polishing. Quantitative evaluation using IOS data at 1 and 2 years postoperatively revealed minor, clinically acceptable wear on the maxillary canine. This case demonstrated successful canine guidance and posterior disclusion, emphasizing the value of digital monitoring as part of long-term maintenance. Finally, the increased number of clinical visits and the requirement for specific digital equipment and software should be acknowledged as limitations. Moreover, since the virtual articulator simulates average mandibular movements, future studies should explore approaches to more accurately replicate patient-specific functional dynamics within the digital workflow.

## CONCLUSION

The present case demonstrated that the use of a digitally guided flowable RC injection technique enabled the precise transfer of simulated tooth morphology into the oral cavity to achieve both esthetic and functional outcomes. Furthermore, the incorporation of a digital workflow may reduce operator dependency and offer restorations with consistent reproducibility.

### Acknowledgments

This research was supported by the Grant-in-Aid for Scientific Research, Grant Numbers 23K09202 from the Ministry of Education, Culture, Sports, Science and Technology of Japan and Research Cluster program of Tokushima University, grant number 2402003. We are grateful to Mr. Kohei Kamoi, a dental technician at Tokushima University Hospital, for his valuable advice and cooperation in the prosthetic work and design of this case.

### Clinical Relevance

The digital workflow for RC injection improves restoration accuracy and reproducibility while minimizing invasiveness. This technique enhances treatment efficiency and predictability, making it a valuable approach in post-orthodontic restorative procedures.

**Fig 2a to d:** Preoperative view of the left maxillary and mandibular canines exhibiting tooth wear. (a) Lateral view – left side. (b) Left lateral movement. (c) Left maxillary canine. (d) Left mandibular canine.

**Fig 1a to d:** Overview of the dentition before orthodontic treatment: (a) Lateral view, left side. (b) Frontal view. Overview of the dentition at the one-year follow-up after the start of orthodontic treatment: (c) Lateral view, left side. (d) Frontal view.

**Fig 4a to c:** (a) and (b) Fabrication of a clear silicone index on a 3D-printed model generated from a digital wax-up. (c) Access hole for resin composite injection.

**Fig 3a to f:** Fabrication of digital wax-up. (a) Lateral view, left side. (b) Reproducing anatomical morphology. (c) Mounted on the virtual articulator. (d) Left lateral movement. (e) Digital wax-up for lateral movement. (f) Views of each surface after digital wax-up.

**Fig 6a to c:** Clinical application of the flowable resin composite injection technique for the mandibular left canine. (a) Left mandibular canine under relative isolation. (b) Placement of the index followed by composite resin injection. (c) Final restoration of the left mandibular canine.

**Fig 5a to c:** Clinical application of the resin composite injection technique for the maxillary left canine. (a) Left maxillary canine after rubber dam isolation. (b) Placement of the index followed by composite resin injection. (c) Final restoration of the left maxillary canine.

**Fig 9a and b:** Comparison of intraoral scanner data between one year and two years after restoration. (a) Maxillary left canine. (b) Mandibular left canine.

**Fig 8a and b:** Overview of the dentition before restoration and at the two-year follow-up. (a) Frontal view of the dentition before restoration. (b) Frontal view of the dentition at the two-year follow-up.

**Fig 7a to d:** (a) and (b) Preoperative and postoperative progress during left lateral movement. (c) Two-year follow-up. Slight marginal discoloration on the mandibular canine. (d) Discoloration removed by polishing.


















